# An implementation framework for planning an RSV immunization program for infants using the long-acting monoclonal antibody

**DOI:** 10.3389/fpubh.2025.1585202

**Published:** 2026-02-10

**Authors:** Jody Tate, Elena Bozzola, Michelle Fiscus, Jaime Pérez-Martín, Manuel Sánchez Luna, Catherine Weil Olivier, Taylor Morris

**Affiliations:** 1 The Health Policy Partnership, London, United Kingdom; 2 Italian Pediatric Society, Rome, Italy; 3 Association of Immunization Managers, Rockville, MD, United States; 4 Murcia Health Department, Murcia, Spain; 5 Neonatology Division, Hospital General Universitario Gregorio Marañón, Madrid, Spain; 6 Pediatrics Department, Paris Cité University, Paris, France

**Keywords:** respiratory syncytial virus (RSV), immunization, immunization programs, implementation, long-acting monoclonal antibody

## Abstract

Respiratory syncytial virus (RSV) infects most children by the age of two. Although the majority experience mild cold-like symptoms, RSV can lead to serious lower respiratory tract infections (LRTIs), including bronchiolitis and pneumonia; the virus is responsible for 3.6 million hospital admissions in children under five globally. More than 70% of RSV-related hospitalizations in children under 1 year of age occur in infants who are otherwise healthy and born at term. Two immunizations have recently been approved with the aim of protecting all infants from severe RSV-related illness: a maternal vaccine, RSVpreF, and a long-acting monoclonal antibody (mAb), nirsevimab. The universal use of a long-acting mAb is a novel approach to disease prevention. It therefore requires careful planning to prepare health systems for widespread delivery. We developed a framework to encourage evidence-based planning for the implementation of RSV immunization programs using the long-acting mAb; the framework can also be used to guide improvements in countries where implementation has already begun. The framework was developed based on a pragmatic review of the published literature as well as the authors' perspectives and early experiences of implementation. It is organized into five domains, based broadly on the World Health Organization's health system building blocks: governance and leadership, reimbursement and funding, demand, service provision, and monitoring and assessment. This article provides an overview of the five domains of the framework, and examples of elements that should be considered under each.

## Introduction

Respiratory syncytial virus (RSV) infects most children by the time they reach their second birthday (1). While many children with RSV experience mild cold-like symptoms, RSV can lead to serious lower respiratory tract infections (LRTIs), including bronchiolitis and pneumonia (1). The virus places a significant burden on health systems; in 2019 it was responsible for 3.6 million hospital admissions [uncertainty range (UR) 2.9–4.6 million] and 101,400 deaths (UR 84,500–125,200) in children under five (2, 3). More than 70% of RSV-related hospitalizations in children under 1 year of age occur in infants who are otherwise healthy and born at term (1, 4, 5).

Monoclonal antibodies (mAbs) work by mimicking antibodies produced naturally in the body. While they have been used for many years as a treatment for diseases including breast cancer and rheumatoid arthritis, their use as an immunization is relatively recent (6). MAbs have been used as an immunization to prevent COVID-19 and RSV in some countries, with more preventive mAbs in the pipeline including for malaria and yellow fever (7).

Given the potentially significant public health benefits, and in response to high levels of RSV-related hospitalization, especially during the 2022/23 RSV season, some countries began implementing immunization programs for all infants at the beginning of the 2023/24 season. Real-world data from France, Spain and the US demonstrated that nirsevimab significantly reduced hospitalization from RSV-related illness by 82%−98% (8–11). Demand among parents was high, with acceptance of the long-acting mAb being at least 80% in France and Spain (11, 12). A number of studies from high-income countries have also found that the long-acting mAb could be cost effective (13–16).

The universal use of a long-acting mAb is a novel approach to disease prevention (Table 1). While one RSV long-acting mAb is already available in many countries (nirsevimab), other products are in development, including one (clesrovimab) that was recently approved for use in the US but has not yet been implemented (17). The introduction of long-acting mAbs therefore requires careful planning to prepare health systems for widespread delivery (18). Some high-income countries started implementing all-infants RSV immunization programs in 2023; others are yet to deliver such programs, and may be considering how best to plan for implementation. Regardless of existing experience, countries need to draw on all of the available evidence to plan and deliver effective immunization programs. To assist with this, we have developed an implementation framework.

**Table 1:** Comparison of two recently approved immunizations which are being implemented in different countries for protecting a broad infant population against severe RSV-related illness.

**Name and type of immunization**	**Indication and recommendations**	**Efficacy**
RSVpreF maternal immunization	Approved in Europe for use between 24 and 36 weeks of pregnancy (46)	81.8% (99.5% confidence interval [CI], 40.6 to 96.3) in preventing medically attended, severe RSV-associated LRTIs within 90 days of birth, reducing to 69.4% (97.58% CI, 44.3 to 84.1) by 180 days after birth (47)
	Some countries, including the United States (US), recommend administration between 32 and 36 weeks (48–50)	
Nirsevimab long-acting monoclonal antibody	Indicated for all infants entering their first RSV season and for infants at increased risk of severe illness who are entering their second RSV season (30, 51)	82.7% (95% [CI], 67.8 to 91.5; *P* < 0.0001) up to 180 days after the dose was given, based on RSV-related LRTI hospitalizations (52)
	Typically recommended for administration as soon as possible after birth for infants born during RSV season, ideally in hospital before discharge. For infants born before the season, it should be given close to the start of RSV season (12, 22, 53)	

## Implementation framework

### About the framework

The “Monoclonal antibodies for RSV implementation framework” is rooted in implementation science; it aims to systematically bridge the gap between evidence and delivery to support effective implementation (19).

The framework is organized around five domains, which broadly align with the World Health Organization's health system building blocks. The framework was developed based on a pragmatic review of the published literature on RSV and immunization programs (see the Supplementary material S1 for a summary of the methodology). The literature review was supplemented by the authors' perspectives and early experiences of implementation. The authors met as a group twice to refine drafts of the framework before it was piloted. Piloting involved applying the framework to three countries that were among the first to implement long-acting mAb immunization programs: France, Spain, and the US. Following the pilot stage, the framework was updated and finalized.

The framework can be used by policy- and decision-makers, researchers, clinicians, and patient advocates to encourage evidence-based planning for the implementation of RSV prevention programs using the long-acting mAb for all infants, adapted to local contexts. It can be applied to countries that have not yet implemented immunization programs, to support preparation. It can also be adapted and used in countries where implementation has already begun, to assess progress, identify gaps and plan for improvements. As such, the implementation framework is applicable to all countries; however, the assessment indicators in each domain would need adapting to reflect the local context (such as available infrastructure and funding), particularly in low- and middle-income countries.

In the following sections, we provide an overview of the five domains of the framework along with examples of elements that should be considered under each. The full framework can be found in the Supplementary material S1.

### Domain 1: governance and leadership

For countries seeking to implement the long-acting mAb for the first time, it is necessary to understand whether it will be evaluated by a national immunization technical advisory group (NITAG) in the same way as a vaccine. Review by a NITAG can bring benefits such as inclusion in a national immunization schedule and a clear path for funding (18). Given that the long-acting mAb is not a vaccine—it does not produce an antibody response, but instead provides passive immunization through the direct administration of antibodies—the scope and responsibilities of the NITAG may need to be adjusted. In the US, for example, the scope of the NITAG [the Advisory Committee on Immunization Practices (ACIP)] had recently been amended to include preventive antibody products (20, 21). This meant that the long-acting mAb was designated a recommended immunization (22). Under the provisions of the Affordable Care Act, US insurance companies are required to cover the cost of ACIP-recommended vaccinations. The inclusion of the long-acting mAb in the Vaccines for Children program made it available to uninsured, underinsured and Medicaid-eligible infants as well as infants who are American Indian or Alaska Native (23, 24).

Engaging multidisciplinary stakeholders including government bodies, clinical societies and patient representatives to make consensus-based recommendations for an all-infants immunization program is important for effective and continued implementation. Strong collaboration and support were important in rolling out early implementation in some countries. In Spain, for example, the Spanish Association of Pediatrics' Advisory Committee on Vaccines recommended the administration of the long-acting mAb to all infants under 6 months of age before it had been recommended by the Public Health Commission (25). This encouraged government bodies to undertake the necessary assessments, resulting in the recommendation to implement an immunization program for all infants for the 2023/24 season and facilitating early procurement. Continued support, including by the Spanish Society of Neonatology, contributed to the successful continuation of the program (25–27).

### Domain 2: reimbursement and funding

Policymakers will need to make sure the immunization program is funded in a way that secures equitable access for all infants. Several factors must be considered, including how reimbursement will work in both hospital and outpatient settings; where the budget will come from in the short to medium term; and how to avoid exacerbating inequalities (e.g., between those using private vs. public insurance). In Spain, for example, there were limited cost-effectiveness data, but health authorities recognized the importance of preventing another severe RSV season. The recommendation for the immunization program using the long-acting mAb was made at a national level initially just for the 2022/23 RSV season, following which all regional health authorities agreed to fund it (28, 29). This meant the product was available and free at the point of delivery for all infants in Spain, supporting broad and equitable access, and resulting in high uptake (30). Other countries have used different mechanisms to support rapid access for families at no additional cost. In France, for example, a direct-purchase model was used during the 2023/24 season to ensure that no payment was required from parents or providers (31, 32). In Ireland, access was accelerated for the 2024/25 season via the “pathfinder” initiative, which seeks to integrate innovative approaches to improving health outcomes (33).

Planning for immunization programs using the long-acting mAb should also consider how to implement them in a way that incentivizes administration, or at the very least does not disincentivize it. In the US, for example, during the first season of implementation, funding did not cover all costs associated with delivering the immunization. This meant that many birthing hospitals were disincentivized from participating. Whereas, in Germany, additional remuneration was endorsed for office-based healthcare professionals. This remuneration covers the costs associated with healthcare professionals providing information and advice to parents, as well as administering the immunization (34).

### Domain 3: demand

A successful immunization program for an innovative approach such as the long-acting mAb requires health authorities to build awareness. Communication campaigns tailored to the local context, and to the public's knowledge and beliefs, are critical to ensuring widespread understanding of the seriousness of RSV and the benefits of immunization. For example, in France where vaccine confidence is relatively low compared to other countries in Europe (35) the long-acting mAb was presented as a preventive therapy rather than a vaccine, whereas in Spain, where vaccine confidence is high (35), it was usually discussed as a vaccine. In both countries, communication campaigns focused on bronchiolitis rather than RSV, as the former was better understood by parents. These strategies were effective, and the acceptance rate for the long-acting mAb was above 80% in both countries (11, 12).

Healthcare professionals involved in recommending and administering RSV immunizations require training and educational resources to fully understand and counsel parents. This information must be clear and shared proactively. In Spain, for example, regional health authorities offered training and developed materials including webinars, web pages and leaflets (36, 37). In countries where the long-acting mAb and maternal immunization are both available, healthcare professionals must be provided with clear information so they can appropriately counsel pregnant people as to their options for RSV prevention. In France, the Haute Autorité de Santé published a press release to complement the formal recommendation to introduce maternal immunization alongside the long-acting mAb. It summarized the evidence and practical considerations for implementation, and included a table listing the benefits and disadvantages of each option (38).

Once an immunization program has been implemented, it will be critical to understand which actions are needed to maintain or increase demand based on ongoing data collection. Continued awareness campaigns and sharing of real-world evidence on the impact and safety of the product can also help reassure parents and support their decision to take up the immunization.

In addition, health system planners will need to make sure that adequate supply is available to meet demand; that demand is monitored; and that supply is adjusted as needed on an ongoing basis. In countries where an immunization program using the long-acting mAb is being implemented for the first time, demand estimates should be based on all available evidence. While this evidence may include uptake of existing childhood immunizations, these data alone are not sufficient. In France and the US, demand for the long-acting mAb was much higher than expected, which led to temporary shortages (39, 40). Data on long-acting mAb uptake in countries that have already implemented immunization programs, as well as national data on parental intent to immunize if available and which are collected in the US, for example should also be considered (41).

### Domain 4: service provision

RSV immunization should be timed to RSV season which, in temperate climates, is typically during the winter months, and ideally delivered at birth for infants born during the season and just before the season for infants born before. As such, administration may take place across a range of inpatient and outpatient settings, depending on the age of the infant. Health system planners must therefore ensure all infants can seamlessly access immunization at the right time, including those who do not receive the long-acting mAb at birth. Systems should be in place to identify and follow up with these infants so that immunization can be provided in outpatient settings.

Careful workforce planning and decisions regarding which healthcare professionals are authorized to deliver the immunization are key to ensure timely administration of the long-acting mAb. Expanding administration to midwives and nurses, as implemented in France and Spain, can increase capacity and support catch-up campaigns for infants born before RSV season. Leveraging existing pediatric vaccination infrastructure can maximize efficiency and reduce the need for additional training and resources, as demonstrated in Andalucía, Spain, where integrating pediatric nursing into primary care during the COVID-19 pandemic facilitated the successful roll out of the RSV immunization program (42, 43).

Given the range of potential delivery settings, robust immunization information systems (IIS) are essential. These systems can support healthcare professionals in confirming infants' eligibility and accurately recording immunization delivery. In countries where both the maternal immunization and the long-acting mAb are recommended, consideration should be given to how medical information is shared between settings so that healthcare professionals can determine whether an infant needs the long-acting mAb or is already protected by maternal vaccination.

### Domain 5: monitoring and assessment

Data on RSV epidemiology are needed to guide the effective planning and implementation of RSV immunization programs. Ideally, RSV surveillance data should be based on virological testing, and may be collected through bespoke systems or as part of existing influenza-like-illness registries. These data can be used to identify when RSV season begins and ends, and measure the burden of RSV in different population groups (44). Real-world data on acceptance and the safety and impact of immunization in reducing RSV cases and associated hospitalizations are also important. These data help health systems adapt future prevention and response strategies, and support public awareness and confidence about the importance of preventing RSV in infants. In Galicia, Spain, real-world evidence collected through studies during the 2022/23 RSV season showed that the immunization program prevented a significant number of RSV-related complications and hospitalizations, and that the product was safe (10). This evidence informed planning and the decision to continue the program with the long-acting mAb for subsequent RSV seasons, and was communicated on a weekly basis to reassure the public and healthcare professionals (10, 27, 45).

## Discussion

Effective implementation of an RSV immunization program for all infants using the long-acting mAb requires a comprehensive understanding of the wider health system context and factors that could impact delivery (Table 2). Social and behavioral science approaches—such as validated surveys—can help assess public confidence in RSV immunization and identify strategies to strengthen it.

**Table 2:** The implementation of all-infant immunization programs using the long-acting mAb: health system challenges and how to address them.

**Domain**	**Challenges**	**Recommended solutions**
Governance and leadership	Limited recognition of the importance of immunizing infants against RSV	Build multidisciplinary support for consensus-based recommendations
	The long-acting mAb may be ineligible for inclusion in vaccination programs	Assess the long-acting mAb in the same way as a vaccine and include it in national immunization programs
Reimbursement and funding	Out-of-pocket costs for parents may be a barrier to uptake	Minimize out-of-pocket costs to help ensure equitable access
	Lack of incentives deters facilities from administering the long-acting mAb	Ensure appropriate incentives for healthcare professionals to recommend and administer the long-acting mAb
Demand	Lack of public awareness about the potential seriousness of RSV	Run targeted communication campaigns, led by health authorities, reflecting parents' knowledge of RSV and attitudes toward immunization
	Healthcare professionals not clear about the benefits of immunization and how/when to deliver available options	Provide training for healthcare professionals on RSV immunization options, schedule and communication with parents
	Demand estimates may be difficult in the early years of implementation	Draw on available data and other countries' experiences to anticipate demand
Service provision	Lack of clarity about when and how to deliver RSV immunization strategies, especially where more than one option is available	Build logistical plans and protocols to support the timely delivery of the long-acting mAb, leveraging existing infrastructures where possible
	Limited workforce to deliver the long-acting mAb catch-up campaign	Boost workforce capacity to implement a rapid catch-up campaign before the start of the season, and involve a range of healthcare professionals
	Lack of systems to record and share data on immunization uptake between settings	Use immunisation information system (IIS) that record administration of the long-acting mAb and maternal immunization; share the immunization status of mother and infant with healthcare professionals
Monitoring and assessment	Gaps in data on the uptake and impact of the long-acting mAb	Collect and use real-world evidence on the uptake, safety and impact of the long-acting mAb to support decision-making and build confidence
	Limited epidemiological data indicating the starting and ending time of the RSV season	Implement real-time RSV surveillance systems to monitor RSV epidemiology and inform the timing of immunization campaigns

To ensure equitable protection of all infants, health system planners must continuously review emerging data and adapt programs to sustain high uptake of RSV immunization. By using the implementation framework on an ongoing basis, policymakers and health system planners can continue to identify and anticipate potential issues or gaps and optimize immunization programs.

## Limitations

The authors took a pragmatic approach to identifying the literature rather than conducting a full systematic review. The authors recognize that some literature may have been missed due to the pragmatic approach taken. While it is possible that the methodology may have affected the robustness of some of the conclusions, we feel this is unlikely given the authors' many collective years of experience in implementing immunization programs, including with the long-acting mAb. The full scope and methodology can be found in the Supplementary material S1.

The framework was developed based on the experience of implementing immunization programs in three high-income countries. The authors are aware that the framework would require significant adaptation for low- and middle-income countries, especially for the former, which likely have very different immunization policy and implementation landscapes.

As declared in the associated conflict of interest statement, most authors of this article previously received funding from the life sciences industry. Although they were not paid to participate in this project, we recognize that these links, alongside our close ties to nirsevimab implementation programs, may have influenced our perspectives. We have built on the literature and made every effort to present our balanced and accurate perspectives as healthcare professionals and health system leaders.

## Data Availability

The original contributions presented in the study are included in the article/Supplementary material, further inquiries can be directed to the corresponding author.
