# Supplementary material for: An implementation framework for planning an RSV immunization program for infants using the long-acting monoclonal antibody
Source: Front Public Health. 2026 Feb 10;13:1585202. doi: 10.3389/fpubh.2025.1585202 (PMC12970533; doi:10.3389/fpubh.2025.1585202)
Supplement: Supplementary file 1 [file Data_Sheet_1.pdf]

frontiers

Supplementary Material

Supplementary Material 1. RSV mAb implementation framework

1 Governance and leadership

1.1 Recommendations and approvals

Are regulatory approval processes well suited to the mAb for RSV prevention?

**Assessment indicators**	• Is there an established regulatory pathway through which the mAb can be assessed?<br>• Is the mAb classified as an immunisation or a therapy by national regulatory bodies?<br>○ How will this affect access?<br>• Which body is responsible for providing regulatory approval for the mAb?<br>• What evidence is required to receive regulatory approval for the mAb? Does this differ depending on how it is classified?<br>• Are the required data available for assessment through the designated pathway?

What is the appropriate process for reviewing and making recommendations for the use of mAbs for RSV in all infants?

**Assessment indicators**	• Will the mAb be evaluated by a National Immunisation Technology Assessment Group (NITAG)?<br>• If it will not be evaluated by a NITAG:<br>○ Will it be evaluated by another established national body with clear assessment procedures?<br>○ How will assessment by a different government body affect implementation (e.g. in terms of funding and access)<br>○ Is there a legal barrier to NITAG evaluation?<br>• Will the mAb be included in a national immunisation programme?<br>○ What factors could facilitate the inclusion of the mAb in a national immunisation programme?<br>○ To what extent does the inclusion in a national immunisation programme influence funding, delivery and coverage?

1.2 Leadership and support

Is there national and/or regional leadership and political support for implementation of an all-infants immunisation programme with the RSV mAb?

**Assessment indicators**	• Are government bodies (e.g. ministry of health, regional health departments) in favour of funding and delivering the mAb for all infants?<br>o If not, what are the reasons and how can their concerns be addressed?<br>• Do relevant public health and health service delivery bodies agree on the need to implement an all-infants immunisation programme with the RSV mAb?<br>o If not, what are the reasons for disagreement and how could it affect approval and implementation of the mAb?<br>• Are all relevant disciplines – including clinical professional societies, public health bodies and health service delivery bodies – collaborating to call for the implementation of an all-infants immunisation programme with the RSV mAb?<br>o What strategies do they use?<br>o If some of these groups are not supportive of the mAb, how might this affect implementation and are there any strategies that could encourage acceptance?<br>• Are patient organisations and professional societies actively involved in advocating for an all-infants RSV immunisation programme?<br>o What strategies do they use?<br>• Which national or regional bodies influence immunisation policy and service delivery, and in what ways?

1.3 Clinical guidelines

Have clinical guidelines been developed for the delivery of the mAb in all settings?

**Assessment indicators**	• Have clinical guidelines been developed to specify:<br>o which ages are recommended to receive the mAb, and how the recommendations differ for high-risk groups? (e.g. annual doses until age two for infants with conditions that put them at greater risk of severe illness from RSV)<br>o when and where infants should receive the mAb at birth and when and where they should receive it as a catch-up? (i.e. to align with the RSV season)<br>o the correct dose that should be given, based on the infant's weight?<br>o any contraindications for use of the mAb, including exposure to the maternal immunisation?<br>o recommendations on possible co-administration with other vaccines?<br>• Which national bodies or professional societies are responsible for developing and updating immunisation guidelines?<br>• How will clinical guidelines impact or support implementation?<br>• Has an RSV immunisation target rate been officially defined, communicated and monitored?

2 Reimbursement and funding

Have the necessary analyses been conducted to inform reimbursement decisions?

**Assessment indicators**	• Has a health technology assessment or other relevant assessment of cost-effectiveness been conducted?<br>o If yes, what was the outcome?<br>o If not, which data and processes are needed to complete such an assessment?

Will reimbursement/payment policies support delivery of the mAb according to national or regional recommendations?

**Assessment indicators**	• How will reimbursement/payment decisions for the mAb be made?<br>o Will the mAb be assessed and funded as a treatment or a vaccine?<br>o Who is responsible for the mAb programme budget planning and when?<br>o Will the necessary funding come from hospital budgets, public health budgets or others?<br>• What is the process for reimbursing/paying for the mAb in hospital settings?

	• What is the process for reimbursing/paying for the mAb in primary care?<br>• Will healthcare providers and hospitals be reimbursed/paid for delivering the mAb to all eligible infants?<br>• Will the mAb be delivered without requiring an out-of-pocket payment (even if this will later be reimbursed)?<br>○ Would upfront payment or copayment be a barrier to access?<br>• Will health insurers (public and/or private) cover any costs of the mAb that are not reimbursed/paid by a national health system or government-funded immunisation programme?<br>• Would out-of-pocket costs be a barrier to access for some families or populations?

Will providers be adequately incentivised to offer and deliver the mAb?

**Assessment indicators**	• How will delivery of the mAb at birth be billed and/or paid?<br>○ Will the cost of the mAb be bundled with all other costs charged for birth and neonatal care, potentially adding a cost to the delivery of maternity care for hospitals?<br>• Will payers provide sufficient reimbursement to incentivise hospitals and/or individual clinicians to offer and deliver the mAb?<br>• Where healthcare providers purchase the mAb independently, will adequate and timely reimbursement be guaranteed to encourage participation in the programme?<br>• Are there performance-based financial incentives to reach RSV immunisation targets?

3 Demand

3.1 Epidemiology and burden of RSV

What is the typical burden of RSV infection in infants under 12 months of age?

**Assessment indicators**	• How many cases of RSV are there in infants each year?<br>• How many hospitalisations and outpatient appointments are attributed to RSV infection in infants and young children each year?<br>• How does RSV infection contribute to seasonal pressures on health systems?<br>• What is the estimated economic cost of RSV infection among infants?<br>• Are there any groups that are disproportionately affected by RSV infection?

	• What is the impact and burden of the longer-term health and social consequences of RSV infection?

How many infants are expected to receive the mAb each year?

**Assessment indicators**	• How many babies are born each year in each region/area?<br>• What proportion of infants will weigh ≥5.0kg when they are eligible to receive the mAb?*<br>• How are the start and end of the RSV immunisation campaign defined and communicated?<br>• What proportion of parents are expected to accept the mAb for their infant?<br>• Are there any populations that are less likely to access or accept the mAb?<br>• How will demand for the mAb be estimated, and which national or regional body is responsible for making this estimate? How far in advance is demand estimated?<br>• How will demand for the mAb be affected by the introduction of maternal immunisation?<br>• What is the ordering process and timeline at national, regional and/or hospital/pharmacy level?

*Infants weighing <5.0kg require a 50mg dose and infants weighing ≥5.0kg require a 100mg dose, so quantities of each dose will need to be ordered.

3.2 Awareness and information

Is appropriate information about RSV and the mAb readily available for expectant parents and parents of young children?

**Assessment indicators**	• What factual, up-to-date information about RSV and the mAb – produced by trusted government organisations, professional societies or patient organisations – is available and accessible for parents?<br>• When and how often is information about the mAb shared through public education campaigns?<br>• What communication channels may be most effective in reaching parents of infants?<br>• Which healthcare professionals will be actively involved in educating parents about RSV and the mAb both before and after birth?<br>• Does the information refer to RSV infection in an understandable way (e.g. by using the most familiar terms related to RSV)?

	• Does the information describe RSV mAbs in the most effective way (e.g. by referring to them as an immunisation or preventive treatment, according to local attitudes and preferences)?<br>• What information is available in the relevant languages and in a culturally sensitive way to meet the needs of local populations to ensure equity in access?<br>• What data are available about public awareness of RSV and/or mAbs, and about public attitudes towards these topics? How can this information be used to help design awareness campaigns?<br>• What are the existing public information or education strategies that can be leveraged to raise awareness and understanding of RSV and the mAb?<br>o Who is involved in delivering these strategies?

Are all relevant healthcare professionals trained and educated on the importance of RSV prevention and accepting of the mAb?

**Assessment indicators**	*Please refer to Table 1A in the Appendix for relevant healthcare professionals.*<br>• What evidence is available to indicate that relevant healthcare professionals understand what the mAb is and that they are aware of the available safety and efficacy data?<br>• What is the evidence (e.g. from surveys or letters of support from professional societies) that relevant healthcare professionals agree that RSV prevention, and the implementation of an immunisation programme using the mAb, should be prioritised?<br>• How will all relevant healthcare professionals receive role-specific training or education on RSV prevention, immunisation delivery and public education strategies?<br>• Which scientific or professional societies are involved, or should be involved, in raising awareness of the mAb among healthcare professionals and in providing relevant training?<br>• What may be the best communication channels for reaching the relevant healthcare professionals?

4 Service provision

4.1 Organisation and logistics

Are plans in place to ensure the mAb immunisation programme will be organised and delivered to all eligible infants?

**Assessment indicators**	• How will the mAb programme be implemented consistently and equitably across the country?<br>• What plans are in place to specify:

	◦ at what point, and in what setting, babies born during RSV season will be offered and receive the mAb?<br>◦ at what point, and in what setting, babies born outside of RSV season will be offered and receive the mAb (e.g. existing routine visits)?<br>◦ how parents of eligible infants will be identified and invited to receive the mAb at the start of RSV season?<br>◦ what strategies will be put in place to make the mAb accessible for underserved populations and to support uptake?<br>• How do plans allow for greater demand and an increase in workload for healthcare professionals at the beginning of RSV season, when the catch-up campaign for babies born out of season is delivered?<br>• Which person or group is responsible for developing these plans and coordinating their implementation?<br>• Which existing processes or pathways can be utilised to support the delivery of the mAb in hospitals?<br>• Which existing health check appointments, processes or pathways can be utilised to support the delivery of the mAb in primary care or community settings?

How will the mAb be transported and delivered to the healthcare facilities that require it?

**Assessment indicators**	• What is the planned logistical procedure for ensuring the mAb doses are transported from the supplier to each healthcare facility in a timely manner for the RSV season?<br>• Which body will be responsible for managing the order and transport of the mAb and ensuring each healthcare facility receives the required number of doses?<br>• How will healthcare facilities communicate with the responsible body about supply of, and demand for, the mAb?

How will maternal immunisation affect the implementation of an infant mAb immunisation programme?

**Assessment indicators**	*Complete this section if maternal immunisation against RSV is available or planned to be rolled out in this country.*<br><br>• Is the mAb or maternal immunisation preferred or prioritised in national recommendations and guidelines?<br>• What proportion of pregnant people receive/are expected to receive the RSV vaccine?

	• What is the guidance for administering the mAb in infants whose mothers were immunized?<br>• How are healthcare professionals informed on how and when to use the mAb or maternal immunisation to prevent potential overlap and administration errors?

4.2 Health system capacity

Are healthcare facilities equipped to store and administer the mAb?

**Assessment indicators**	• Do all healthcare facilities implementing the mAb immunisation programme have the necessary equipment and systems in place? This includes:<br>○ procedures for prescribing and documenting the administration of the mAb<br>○ equipment needed for giving injections, including sharps disposal containers, biohazard bins and personal protective equipment<br>○ equipment needed for the proper storage of the mAb and staff who are trained in the cold chain requirements (which are the same as for other vaccines).<br>• Will all doses be delivered at the beginning of RSV season, or will they be stored centrally and allocated to healthcare facilities on a rolling basis throughout RSV season?<br>• What are the plans for collecting and returning unused or expired doses at the end of the campaign?

Are adequate data-sharing systems in place to support implementation of the mAb immunisation programme across different sites?

**Assessment indicators**	• Is there an electronic data-sharing system in place that can be accessed by hospitals and primary care centres to identify infants who have received the mAb and (if relevant) whose mothers received the maternal RSV vaccine?<br>○ If not, is there any systematic way for providers across the healthcare system to identify which infants have received the mAb (or passive immunisation through maternal RSV immunisation)?<br>• Can existing data-sharing systems be used to collect data about access and uptake of the mAb?<br>• Are data systems or electronic health records used to:

	o trigger reminders for healthcare professionals to offer the mAb, or for parents to request it?<br>o identify and recall high-risk infants to receive the mAb in their second and subsequent RSV seasons (as directed by national guidelines)?<br>• Is data sharing a priority for the health system more broadly?

4.3 Workforce capacity

Do hospitals and primary care centres have sufficient workforce capacity to deliver the mAb to all infants during RSV season?

**Assessment indicators**	*Please refer to Table 1A in the Appendix for a list of relevant healthcare professionals who should be considered.*<br><br>• What are the existing workforce-related challenges in delivering neonatal or postnatal services that suggest the implementation of a new immunisation programme could be difficult?<br>• Which healthcare professionals are allowed to administer intramuscular injections in infants?<br>• Is there a known shortage of primary care staff that could affect the feasibility of delivering the mAb to newborns during RSV season or as a catch-up programme in the autumn?<br>• What are the attitudes and beliefs of healthcare staff about implementing a new immunisation programme for all infants?<br>• Which healthcare professionals are authorised to prescribe the mAb? Which healthcare professionals are authorised to administer it?<br>• Within the wider health system, are there calls to expand the right to prescribe and administer injections to a greater range or number of healthcare professionals, which may be relevant to the mAb?

5 Monitoring and assessment

Is there sufficient data collection on RSV to guide future planning and implementation of the mAb immunisation programme?

**Assessment indicators**	• Is mandatory routine surveillance in place to monitor the incidence, burden and seasonality of RSV?

	o Is virological surveillance in place in both hospitals and outpatient settings to differentiate cases of RSV from influenza and COVID-19, and to monitor the activity of different RSV strains?<br>o Is point-of-care testing used in hospitals and outpatient settings to support RSV surveillance?<br>• Apart from clinical research data, how will the following real-world data on the mAb routinely be collected and reported?<br>o Data on safety and effectiveness.<br>o Data on coverage and uptake, including data on which population groups are more or less likely to access or accept the mAb.<br>o Data on healthcare professionals' and parents' attitudes and experiences with the mAb.<br>o Data on cost-effectiveness, including the cost-effectiveness of different approaches to implementation.<br>• How and when will surveillance and uptake data be updated and publicly shared during RSV season?<br>• Can data collection for RSV infection and the mAb be incorporated into existing data collection and surveillance systems, or will new systems be developed?<br>o Who is accountable for developing, implementing and running data collection platforms?

How will data on RSV and the mAb be used to inform health system planning?

**Assessment indicators**	• Will data on mAb uptake be used to inform demand estimates in subsequent RSV seasons?<br>• Will evidence of the effectiveness of the mAb in preventing hospitalisations be used to advocate for sustained political will to provide and promote the mAb?<br>• Will all collected data on RSV and mAb uptake and impact be made available to health system planners rapidly, or in real time, to enable evidence-based decision-making?<br>• Will there be longer-term follow-up to assess the wider health impacts of preventing RSV in infancy?

Supplementary material 2. Literature review methodology

A. **Scope**

- Disease area: Respiratory syncytial virus
- Specific area: Immunization policies and programs, including maternal immunization and infant immunization

- Publication dates: Documents published between 2019 and 2024 to capture the current landscape
- Population: Infants and young children, with a focus on those aged 2 years and younger
- Geography: High-income countries

B. **Combinations of search terms (first row combined with each subsequent row in turn, using AND)**

**Core concepts**	"Respiratory Syncytial Virus" OR RSV OR bronchiolitis OR "lower respiratory tract infection"	"immunization programs"[Mesh] OR prophyla* OR antibod* OR mAbs
**Implementation**	Implement* OR deliver* OR integrat*	
**Governance**	"Policy"[Mesh] OR "health policy"[Mesh] Or recommendation OR guideline OR guidance OR strategy	
**Regulation and reimbursement**	"Drug approval"[Mesh] OR regulation OR "Insurance, Health, Reimbursement"[Mesh] OR reimburse* OR funding OR budget	
**Demand**	"Cost of illness"[Mesh] "vaccination hesitancy"[Mesh] OR awareness OR uptake	
**Service provision**	Infrastructure OR "workforce"[Mesh] OR training OR hospital OR neonatal OR "primary care" OR "vaccination center" or "immunization center" OR maternity OR postnatal OR pediatric OR obstetric* OR pharmacy OR midwife OR nurse	
**Health information**	"Data collection"[Mesh] OR database OR registry OR hospitali* OR "Patient Reported Outcome Measures"[Mesh]	

C. **Sources of grey literature**

Websites of various organizations in many countries were interrogated for information on RSV and implementation of immunization programs. Examples include:

- American Academy of Pediatrics: https://www.aap.org/
- Centers for Disease Control and Prevention: https://www.cdc.gov/
- ReSViNET: https://resvinet.org/
- International RSV Society: https://www.isrv.global/

- Asociación Española de Pediatría: https://www.aeped.es/
- All regional health authority in Spain
- Nirse-gal: https://www.nirsegal.es/en
- Santé publique France: https://www.santepubliquefrance.fr/

D. **Other techniques to identify literature**

Snowball searches were carried out by scanning the references of included publications for additional papers. Citation searches in Google Scholar were also used to find additional papers that reference included publications.
